# Efficacy and Safety of ab Externo Phaco-Canaloplasty versus First-Generation iStent Bypass Implantation Combined with Phacoemulsification in Patients with Primary Open Angle Glaucoma-Early Results

**DOI:** 10.3390/ijerph20021365

**Published:** 2023-01-12

**Authors:** Kinga Gołaszewska, Iwona Obuchowska, Joanna Konopińska

**Affiliations:** Department of Ophthalmology, Medical University of Białystok, 15-089 Białystok, Poland

**Keywords:** canaloplasty, iStent, POAG, Schlemm’s canal surgery, phacoemulsification, glaucoma

## Abstract

This study evaluated the early outcomes of the hypotensive efficacy and safety profile of ab externo phaco-canaloplasty versus first-generation iStent bypass implantation combined with phacoemulsification in patients with primary open-angle glaucoma (POAG). In total, 82 patients with POAG comprising 92 eyes were divided into phaco-canaloplasty (Group PC, (*n* = 47) or iStent combined with phacoemulsification (Group PiS, *n* = 45) groups. Primary outcome measures were intraocular pressure (IOP) reduction and number of glaucoma medications. Secondary outcome measures were best-corrected visual acuity (BCVA), endothelial cell density (EECD), changes in anterior chamber depth (ACD), and complication rate. The follow-up period was approximately 6 months. Preoperative IOPs were 17.30 (15.00; 19.85) mmHg and 17.50 (15.10; 20.90) mmHg in the PC and PiS groups, respectively (*p* = 0.876). At the end of the follow-up, IOP decreased to 15.00 (13.00; 16.00) mmHg and 15.00 (14.00; 17.00) mmHg in the PC and PiS groups, respectively (*p* = 0.438). Medication usage decreased from 2.08 to 0.12 and 1.40 to 0.04 in PC and PiS eyes, respectively. Most patients in both groups were medication-free at 6 months follow-up. After 6 months of observation, EECD in PC and PiS groups decreased from 2309.50 (2032.00; 2533.00) to 1966.50 (1262.00; 2353.50) and 2160.00 (1958.50; 2372.50), to1231.00 (1089.00; 2050.00), respectively (*p* = 0.037). Pre-surgery BCVA was 0.80 (0.50; 1.00) and 0.60 (0.40; 1.00) in PC and PiS eyes, respectively (*p* = 0.456). Follow-up BCVA was 0.95 (0.60; 1.00) for PC and 1.00 (1.00; 1.00) for PiS. Hyphema and corneal oedema were noted on the first day post-surgery in both groups. Subsequent complications included a transient increase in IOP in the PC group. Phaco-canaloplasty and iStent bypass implantation combined with phacoemulsification significantly lowered IOP and decreased medication burden. All eyes in both groups maintained or exhibited improved BCVA relative to baseline. Both surgeries had low postoperative complication rates and exhibited comparable safety profiles over 6-month follow-up in patients with POAG.

## 1. Introduction

Glaucoma is a leading cause of irreversible blindness and the second leading cause of blindness, leading to a huge global burden. Primary open angle glaucoma (POAG) is the predominant subtype of glaucoma. The number of POAG cases in the adult population (40–80 years old) is estimated to be 120 million individuals in 2040 [1]. This disease results in vision loss due to progressive optic neuropathy resulting from atrophy of retinal ganglion cells, which is associated with characteristic changes in the visual field. Treatment predominantly aims to lower intraocular pressure (IOP) [2], which remains the main risk factor associated with glaucoma progression and visual field deterioration. Several key studies have confirmed the strong association between reduced IOP, glaucoma progression, and vision loss. This was quantified in the landmark Early Manifest Glaucoma Trial (EMGT) as an approximately 10% reduced risk of glaucoma progression for every 1 mmHg reduction in IOP [3]. Failure to adjust IOP can result in progressive, irreversible, and generally painless vision loss. Achievement and maintenance of target IOP reduce the loss of retinal ganglion cells and depend on the balance between the rate of secretion of aqueous humour by the ciliary body and rate of its outflow from the eyeball, 90% of which is drained by the trabecular meshwork (TM) and Schlemm’s canal (SC). The SC is a venous sinus that communicates the anterior chamber of the eye with the episcleral veins through a specialised vascular wall (TM) [4,5,6]. In eyes with POAG, the outflow of aqueous humour is hindered by increased resistance at different levels of the outflow tract. The location of maximum resistance for the aqueous humour is probably the inner wall of SC (TM) [7,8].

In the hypotensive treatment of glaucoma, pharmacological methods are typically used first, followed by laser therapy and surgery. Eye-drop medications often require application more than once a day. Intolerance, incorrect application of drops and compliance may cause treatment failure. Surgical treatment of IOP should be considered when disease progression is observed, despite conservative treatment. To this day, trabeculectomy is the gold standard in the surgical treatment of glaucoma [9,10]. Nevertheless, despite its effectiveness, it is associated with significant complications related to filter bleb formation. This underpins the development of “blebless” procedures, or minimally invasive glaucoma surgeries (MIGS) [11]. However, a reduction in the number of complications due to improvements in surgical methods allows for earlier decision for surgical treatment, resulting in permanent and safe control IOP [12,13] Ab externo canaloplasty belongs to the group of “blebless” procedures [14], whereas iStent is included in the MIGS group [15,16,17]. The common feature of this group of procedures is the lowering of IOP by improving the physiological mechanisms of aqueous humour outflow through the conventional pathway. Aqueous humour passes from the posterior chamber through the pupil into the anterior chamber and exits via two pathways: the conventional or trabecular outflow pathway, or the unconventional or uveoscleral outflow pathway [9]. The conventional pathway continues with humour drainage through the following sequence of structures within the angle of the eye: TM, SC, collector channels, and episcleral venous system. The flow-through of the TM is entirely passive. The flow-through of SC has been demonstrated via paracellular and intracellular pores. Aqueous humour continues flowing through collector channels until it reaches the episcleral venous system, where it is deposited into the systemic cardiovascular circulation [10]. Another IOP-lowering mechanism of canaloplasty is drainage of the aqueous humour through the trabecular-Descement’s window (TDW) into the intrascleral lake, a potential space under the superficial scleral flap which also leads to a reduction in IOP. From the intrascleral lake, the aqueous humour may drain into SC, supravascular space, directly into the sclera, epidural, choroidal canals, and/or subconjunctivally, forming a bleb despite being sealed tightly [11]. The effectiveness of canaloplasty has been compared to trabeculectomy with mitomycin C and deep sclerectomy, with the former procedure having fewer complications compared to traditional methods. Studies on canaloplasty have reported a promising reduction in IOP, with only a few postoperative complications [12,13,14]. The effectiveness of iStent in different populations has been described extensively [15,16,17] and has been compared to various other procedures [18,19,20,21,22]. These studies have reported a mild hypotensive effect with a clear reduction in the burden of pharmacological treatment after the procedure [23,24,25,26,27].

This study aimed to compare the effectiveness and safety of two anti-glaucoma procedures: ab externo canaloplasty and first-generation iStent implantation in combination with cataract phacoemulsification. Although these procedures belong to two different types of anti-glaucoma surgery, they involve the same handling mechanism: improving the outflow of aqueous humour using conventional pathway. Canaloplasty is a procedure in which two scleral flaps are dissected and a suture is introduced into the SC to tighten the canal walls, thereby improving outflow through the TM, into the SC, and subsequently into the collector channels. The iStent-microstent from Glaukos (Glaukos Corporation, Laguna Hills, CA, USA) belongs to the group of MIGS, which, according to the definition, is performed using the ab interno approach through a clear corneal incision without scleral incision and conjunctival dissection. The point of action for this procedure, as in the case of canaloplasty, is the TM, which is the region of greatest resistance to the outflow of aqueous humour. iStent implantation bypasses this site of resistance. Aqueous humour flows through the bypass into the SC and then into the collector channels. To our knowledge, this is the first study to compare these two types of glaucoma procedures.

## 2. Materials and Methods

Data for the study were collected with the consent of the Bioethics Committee of the Medical University of Bialystok (consent no SUB/1/DN/22/003/1157) in accordance with the principles of the Declaration of Helsinki and good medical practice. All patients provided written consent for the surgery, follow-up study, and use of their examinations in scientific publications.

This prospective single-centre trial included consecutive adult patients with POAG who underwent ab externo phaco-canaloplasty or first-generation iStent bypass implantation combined with phacoemulsification between April 2021 and April 2022. This study was conducted at the Department of Ophthalmology Medical University of Bialystok, Poland. All surgeries were performed at a single centre by the same surgeon (J.K.).

A total of 92 eyes (82 patients) with POAG were included in the study, of which 47 eyes were treated with ab externo phaco-canaloplasty, and 45 eyes were implanted with the iStent device combined with phacoemulsification. Inclusion criteria were as follows: 21 years of age, had cataract requiring surgery, one or both eyes diagnosed with mild-to-moderate POAG with inability to obtain the target IOP despite maximally tolerated pharmacological treatment (the result was based on two standardised measurements at least 4 days apart in an eye qualified for surgical treatment). An eye qualifying for the examination had characteristic defects in the layer of nerve fibres in optical coherence tomography examination (OCT, Heidelberg Engeneering). Patients provided informed consent to participate in the study and a declaration of attendance at scheduled control visits. Exclusion criteria were: unwillingness to participate, one or both eyes with vision less than “finger counting”, narrow-angle glaucoma, acute angle closure in the last 12 months, neovascularization, pseudoexfoliation, pigmentary glaucoma, previous laser or surgical treatment.

The primary outcome measures were IOP reduction and reduction in glaucoma medication use. The secondary outcome measures were best-corrected visual acuity (BCVA), endothelial cell density (ECD), changes in anterior chamber depth (ACD), and complications.

The decision to assign patients to the PC and PiS groups was based on the knowledge and experience of the glaucoma surgeon and the anatomical capabilities of the eye.

### 2.1. Preoperative Examination

Patient medical history of glaucoma type, dose of anti-glaucoma medication used, previous ophthalmic treatment, laser treatment, or surgical procedures was obtained at the time of qualification. Before surgical intervention, all patients underwent a baseline standard ophthalmic examination that included BCVA (Snellen’s chart), slit-lamp biomicroscopy for anterior and posterior segment evaluation, gonioscopy with angle grading (Shaffer classification), indirect ophthalmoscopy for fundus assessment including the cup to disc ratio rating, and IOP measurement using Goldmann applanation tonometry between 8 and 10 AM. Cataract grading was performed using the Lens Opacification Classification Scale III (LOCS III). Corneal endothelium was evaluated using an SP-3000P non-contact specular microscope (Topcon Medical Systems 48 Inc., Oakland, CA, USA). The number of endothelial cells per square millimeter (endothelial cell density—ECD) was measured (50 cells were selected each time, if possible). ACD was analysed using Pentacam. Glaucoma severity was diagnosed using standard methods (Humphrey visual field analyser, Carl Zeiss AG, Jena, Germany with the SITA Standard 30-2 algorithm).

### 2.2. Surgical Technique

All patients underwent standard cataract phacoemulsification with implantation of an artificial posterior chamber IOL into the capsular bag. Non-penetrating glaucoma surgery (NPGS) canaloplasty was first described by Stegmann [21]. In brief, ab externo PC surgery was performed under local retrobulbar anaesthesia with 3.5 mL 2% xylocaine. A corneal bridle suture was placed at the 12 o’clock position to rotate the eye inferiorly. The conjunctiva and Tenon’s capsule were opened in the limbus to expose the sclera. Haemostasis cautery was performed if necessary; however, cauterisation was limited where possible to avoid damage to the episcleral veins. A parabolic impression (Kearney marker) was created to outline the superficial flap. The superficial flap (approximately 5.0 × 5.0 mm) was approximately one-third of the scleral thickness between 200 and 250 μm. The deep scleral flap was dissected slightly smaller (approximately 4.0 × 4.0 mm) than the superficial flap and just above the choroidal tissue [11]. The flap was prepared forward into the periphery of the cornea for a length of approximately 2 mm, revealing a window in the trabeculo-Descemet window (TDW). The microcatheter was inserted through one of the cut ends of SC and carefully guided around the perimeter of the canal through fiberscope illumination until the catheter tip was visible at the other end of the canal. Then, a 10-0 polypropylene suture (Prolene, Ethicon Inc., Johnson & Johnson, New Brunswick, NJ, USA) was affixed to the tip of the microcatheter [22] to form a double loop. Viscocanalostomy with additional passage of a microcatheter (iTrack 250A, iScience Interventional, Menlo Park, CA, USA) through SC was performed (360°) with viscoelastic injection every 2 h to dilate the canal, and a loop was created, encircling the inner wall of SC. The suture loop was tightened to distend the TM inwards. The sutures were placed under tension, and locking knots were added [14]. The suture was retained in SC to reduce outflow resistance and increase the permeability of the TM, SC, and juxtacanalicular portion [12]. The superficial flap was sutured tightly with 5 interrupted 10–0 monofilament nylon sutures to prevent leakage and subsequent bleb formation. The conjunctiva was sutured over the limbus using one or two sutures.

The iStent is an L-shaped 0.3-mm wide and 1-mm long stent composed of titanium covered with heparin. The stent ends with a pointed tip to facilitate its insertion into SC, bypassing the site of greatest resistance to the outflow of aqueous fluid from the eye in the IOP-peritubular trabeculae. iStent seton implantation was performed with ab interno access, leaving the conjunctiva and sclera intact. After phacoemulsification under local anaesthesia (Alcaine drops), carbachol was administered into the anterior chamber to constrict the pupil and improve visualisation of the angular structures. After applying a large amount of viscoelastic material to the anterior chamber, which widened the angle of filtration, the patient’s head and operating microscope were rotated 30–45° from the surgeon to better visualise the angle of filtration and facilitate access. The Swan Jacob gonioprism was placed over the eyeball. The implant, placed on a special injector, was inserted into the anterior chamber through the side port during phacoemulsification. When the angle was exposed, the tip of the iStent was inserted into the trabecular at the site of the highest concentration of collector channels (nasolabial quadrants). The appearance of a small amount of blood confirmed the placement of the implant in SC. Aspiration and irrigation were used to remove viscoelastic substances and blood from the anterior chamber. Finally, the tightness of the corneal port was assessed.

### 2.3. Postoperative Visits and Treatment

Examinations were conducted before and after surgery on days 1, 7, 14, and 1, 3, 6 months after surgery. All patients received a topical antibiotic for 2 weeks, topical nonsteroidal anti-inflammatory drugs, and topical steroid for 4 weeks after surgery. Antiglaucoma drops were discontinued on the day of surgery. Antiglaucoma pharmacological treatment was reinitiated if the postoperative target IOP was not achieved.

### 2.4. Statistical Analysis

Statistical analysis was conducted using the statistical program SPSS version 27, with α = 0.05. Dependencies between groups and qualitative variables were determined using the chi-squared test or Fisher’s exact test. Comparisons of quantitative variables between groups were performed with Student’s *t*-test for independent samples or Mann–Whitney’s U test. Comparisons for dependent samples were performed using Wilcoxon’s test or Student’s *t*-test for dependent samples, depending on the normality of the distribution (assessed with Shapiro–Wilk’s test). The confidence intervals for median differences were calculated using the Hodges–Lehmann estimator. Dependencies between measurements and qualitative variables with two categories were analysed with McNemar’s test and Wilcoxon’s test for variables with more than two categories. The sample size was calculated using an online sample size calculator (https://statulator.com/SampleSize/ss2PM.html#, accessed on 28 February 2021). The study would require a sample size of 28 (number of pairs) to achieve a power of 80% and level of significance of 5% (two-sided) for detecting a mean difference of 2.50 between the IOP level at baseline and after 3 months, assuming the standard deviation of the differences to be 4.50. To detect a mean of differences of 3.50 with standard deviation of the differences equal to 5.50, the required sample size (number of pairs) was 23. The assumed range of differences was based on expert knowledge.

## 3. Results

The proportion of women was higher in the PiS group than in the PC group (78% vs. 22%; OR 95% CI = 2.59 (1.04, 6.44); *p* = 0.038). Patients in the PiS group were significantly older than those in the PC group (72.35 vs. 65.11, respectively, *p* = 0.0032; MD 95% CI = 7.24 (2.49, 11.99); *p* = 0.003) (Table 1).

BCVA after 6 months was significantly higher in the PiS group than in the PC group (MD 95% CI = 0.05 (0.00, 0.10); *p* = 0.037) (Table 2). ECD level after 3 and 6 months was significantly lower in the PiS group than in the PC group (MD 95% CI = −437.72 (−733.49, 141.95); *p* = 0.004 after 3 months and MD 95% CI = −735.50 (−784.00, −27.00); *p* = 0.037 after 6 months) (Table 3).

**Figure 1:**
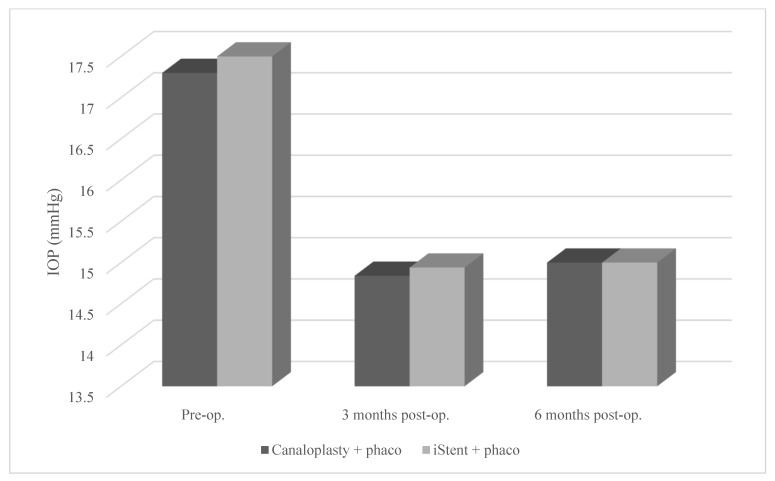
Mean IOP from baseline to 6 months post-surgery according to type of surgery (PC vs. PiS).

**Figure 2:**
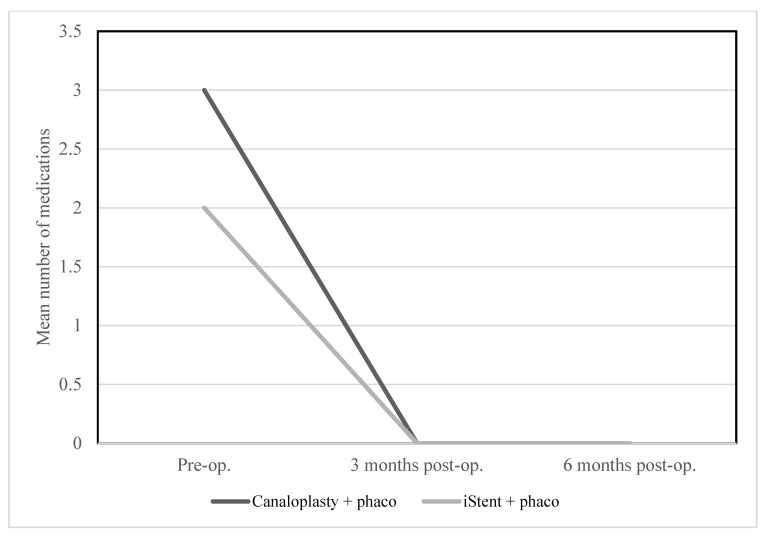
Mean number of medications from baseline to 6 months post-surgery according to type of surgery.

There was no significant dependency between group and number of medications taken at baseline or after 3 and 6 months, glaucoma, follow-up time, BCVA at baseline and after 3 months, IOP level at baseline or after 3 and 6 months, and ECD level at baseline (Table 2, Table 3, Table 4 and Table 5 and Figure 1 and Figure 2).

In the PC group, ECD levels were lower at each time point with sufficient patients than at baseline (*p* < 0.001 for all analyses). The largest difference was observed between baseline and measurements after 3 months, whereby the mean difference was equal to MD = −376.34 (95% CI = −538.36, −214.32). BCVA was higher after 1 month (MD with 95% CI = 0.20 (0.00, 0.15); *p* = 0.039) and after 3 months (MD with 95% CI = 0.25 (0.00, 0.20); *p* = 0.037) than at baseline. IOP level was also lower at each time point with sufficient patients than at baseline (*p* < 0.001). The largest decrease in IOP level was observed after 3 months, with MD = −3.30 (95% CI = −5.35, −2.25). An IOP level ≤ 15 mmHg was observed more frequently after 14 days (54%), 1 month (63%), 3 months (63%), and 6 months (67%) than at baseline (25–31% depending on specific comparison relative to baseline; *p* < 0.050 for the aforementioned analyses). IOP ≤ 18 was also observed more frequently after 14 days (78%), 1 month (91%), 3 months (91%), and 6 months (89%) than at baseline (51–57% depending on specific comparison relative to baseline; *p* < 0.050 for the aforementioned analyses). ACD level was higher at each time point with sufficient patients than at baseline (*p* < 0.050 for all analyses). The largest increase in ACD level was observed after 3 months compared to baseline (MD = 1.38 (95% CI = 0.96, 1.71). At each time point after the procedure (except after 1 year, where *n* = 4), patients were taking fewer medications than at baseline (*p* < 0.001 for all dependencies). After 14 days, all patients (*n* = 37) were not taking any medications (at baseline, 3% of those patients were not taking any medications). After 1, 3, and 6 months, the percentages of patients not taking any medications were 94% (vs. 11% at baseline), 84% (vs. 13% at baseline), and 79% (vs. 14% at baseline), respectively (Table 6).

ECD levels were significantly lower after each measurement than at baseline in the PiS group (*p* < 0.050 for all analyses). The highest difference was observed after 6 months, with an MD level of −1065.00 (95% CI = −955.50, −528.00). A significant increase in BCVA relative to baseline was observed in each measurement up to 6 months, with the largest increase (MD = 0.40) after 1 month (95% CI for MD = 0.05, 0.40; *p* = 0.006) and 6 months (95% CI for MD = 0.10, 0.35; *p* = 0.001). IOP level in the PiS group was lower after 3 months (MD with 95% CI = −2.63 (−4.38, −0.88); *p* = 0.005) and 6 months (MD with 95% CI = −2.40 (−3.30, −0.85); *p* = 0.002) than at baseline. After 3 and 6 months, there were more patients with IOP levels ≤ 15 mmHg than at baseline (62% vs. 28%; *p* = 0.013 for 3 months vs. baseline and 62% vs. 24%; *p* = 0.003 for 6 months vs. baseline). After 6 months, there was a greater proportion of patients with IOP levels ≤ 18 mmHg than at baseline (90% vs. 66%; *p* = 0.016). ACD level was higher at each time point than at baseline (*p* < 0.001 for all analyses), with the greatest difference (MD = 2.16) observed after 6 months (95% CI for MD = 1.40, 2.21). At each measurement after the procedure, patients were taking less medication than at baseline (*p* < 0.050 for all dependencies). After 2 weeks, none of the patients were taking any medications (*n* = 20); at baseline, 1 month, 3 months, and 6 months, one patient, 94% (vs. 12% at baseline), 91% (vs. 9% at baseline), and 96% (vs. 9% at baseline) were not taking any medications (Table 7).

### Intraoperative and Postoperative Complications

PC intraoperative/early postoperative complications included passage of the microcatheter into the anterior chamber that occurred during cannulation (4.7%), microhyphema (<1 mm blood level in the anterior chamber) (38%), hyphema (>1 mm blood level in the anterior chamber) (53%), elevated IOP (5%), and Descemet’s membrane detachment (0.7%). No cases of hypotonia or anterior chamber shallowing were observed (0%).

PC late postoperative complications included posterior capsule opacity (3%) and transient elevation of IOP (3%). Endophthalmitis was not observed, and only a few complications were noted: hyphema (56%) and corneal oedema in both groups on the first day post-surgery; subsequent complications included a transient increase in IOP in the PC group, and two eyes (4.25%) received additional trabeculectomy.

PiS intraoperative/early postoperative complications included microhyphema (<1 mm blood level in the anterior chamber) (11%) and elevated IOP (4.3%).

PiS late postoperative complications included opacity of the posterior capsule (6%), transient increase in IOP (4%), iStent obstruction (4.3%), incorrect placement of the iStent (2.6%), blurred vision or visual disturbances (3.4%), and two cases (4.44%) of bullous keratopathy. No cases of endophthalmitis or hypotonia were noted (0%).

## 4. Discussion

In our study, we achieved a satisfactory hypotensive effect after both PC and PiS, which did not differ between groups. Although canaloplasty works via few IOP-lowering mechanisms, the hypotensive effect is the same as that after iStent implantation, which works only by bypassing the TM. This sheds light on the effect of the transscleral lake, which may not play a key role in the mechanisms underlying the hypotensive effect of canaloplasty. Similar conclusions were drawn by Kicińska et al. [28] in a recent study, in which they compared three variants of canaloplasty: ab externo (ABeC), ab interno (AbiC) and minicanaloplasty (miniAbeC). In their study, no difference was observed between the IOP-lowering effect of the procedure with formation of a transclear lake (AbeC) or variants without the presence of a lake (AbiC and miniAbeC).

Another issue is the effect of phacoemulsification itself on post-surgical decline in IOP (in our study all eyes underwent combined phaco-glaucoma procedures). According to the different studies, it is on average: 1.4 mmHg, 1.9 mmHg, 1.55 mmHg, 1.88 mmHg, 2.9 mmHg, 3.1 mmHg, and 4.9–5.3 mmHg [29,30,31,32,33,34,35,36,37]. Depending on the type of glaucoma, the largest decline in IOP is observed in the eyes with closed-angle glaucoma, and in pseudoexfoliative glaucoma (PXG) (this effect is transient and after a year IOP gradually increases). Analyzing published studies, it can be concluded that the largest decrease in intraocular pressure occurs between the third and sixth month after surgery [34,35]. From our previous studies, this effect remained at a similar level throughout the entire follow-up period, which was 1 year, generally showing the lowest values after half a year of follow-up [36]. Hayashi et al. described an analogous IOP drop of 6.9 mmHg within 12 months after surgery, and even greater, as much as 7.2 mmHg, during 24 months after surgery [37]. Generally, it can be concluded that this effect is most expressed during the first year after surgery [38], although there are reports in the literature that a reduction in IOP was noted even 10 years after surgery [39]. In addition, patients included in our study are qualified for combined glaucoma treatment without a wash-out period before the procedure. Thus, the baseline IOP is at the patient’s maximum tolerated therapy. If such a patient had undergone only cataract surgery, we would not have achieved the target IOP without anti-glaucoma drugs usage. Performing a combined procedure allows us to achieve safe and permanent control of the IOP. Concluding, our patients after combined PC and PiS surgery achieved 2.3 mmHg and 2.5, respectively, lower IOP than baseline in the sixth month of observation but, in most cases, without anti-glaucoma drugs.

Endothelial cells (ECs) are critical structures within the cornea that maintain corneal clarity via their pump function. ECs are naturally lost over time but can be injured medically, surgically, or due to various dystrophies [40]. The potential risk of corneal oedema is 500–800 cells/mm^2^. The borderline density of ECs is approximately 500 cells/mm^2^. Below this value, the cornea decompensates [41]. Surgical treatment of glaucoma carries the risk of corneal endothelial damage due to mechanical manipulation in the anterior chamber of the eye [42]. In our study, 6 months after the procedure, progressive ECD loss was observed in the PC and PiS groups. ECD loss was significantly greater in the PiS group than in the PC group. A greater loss of endothelial cells in the PiS group compared to the PC group may result from a greater number of maneuvers in the anterior chamber performed during the procedure. During the iStent bypass implantation procedure, after phacoemulsification, implantation and appropriate fixation, the IOL carbachol is administered into the anterior chamber, then a large amount of viscoelastic material and, finally, the implant, placed on a special injector, is inserted into the anterior chamber. After iStent implantation, viscoelastic substances and blood are removed from the anterior chamber by the aspiration and irrigation. On the other hand, maneuvers performed in the anterior chamber during the PC procedure are limited to only basic maneuvers during phacoemulsification. Another study conducted by the authors [23], in which combined cataract removal by phacoemulsification with simultaneous iStent implantation was compared with phacoemulsification alone, confirmed the safety of iStent implantation in terms of its effect on ECD for mild-to-moderate open-angle glaucoma. Statistical analysis revealed that iStent implantation did not significantly increase corneal endothelial cell loss compared with phacoemulsification alone in patients who underwent surgery. In our study, of the 45 patients who underwent PiS, only 2 presented with bullous keratopathy as a late postoperative complication; however, in these cases, the preoperative EC count was 1000–1200 cells/mm^2^. One of these patients was treated conservatively, while the other qualified for a corneal transplant due to ineffective local treatment. A review of the literature by Chen et al. [43] did not disclose cases of bullous keratopathy and the need for corneal transplantation. 

Our study has some limitations. First, because of its consecutive nature, the surgeries were not randomly selected. Thus, the baseline characteristics and indications for surgery for each patient might differ between the groups. For example, patients who received Phaco + iStent were older than those who underwent phaco-canaloplasty, because they tended to have concomitant visually significant cataracts. Second, information regarding ultrasound power used during phacoemulsification, which might influence the ECD loss outcome, was not available. However, all procedures were performed by the same surgeon with the same technique following the institute’s protocol, and the grade of cataract did not differ between the groups.

Improvements in anti-glaucoma surgeries, including the emergence of modern methods such as “blebless” and MIGS, has significantly reduced the number of complications, which has facilitated earlier decision to surgical treatment. This results in greater effectiveness for lowering IOP compared to pharmacological methods and circumvents the need for patients to constantly use eye drops. 

## 5. Conclusions

Phaco-canaloplasty and iStent bypass implantation combined with phacoemulsification significantly lowered IOP and decreased medication burden. The PiS procedure is associated with greater ECD loss from the immediate postoperative period, which continues over time at lower rates. The PC procedure is safer in terms of ECD loss. All eyes in both groups maintained or demonstrated improved BCVA compared to baseline. In conclusion, both types of treatment are effective in lowering IOP and medication. The PC has a more favorable safety profile over 6-month follow-up in patients with POAG. This is the first study to compare phaco-canaloplasty with phaco-iStent. Nevertheless, the results should be confirmed by comparative studies or clinical trials, allowing for additional statistical inferences based on a longer period of observation.

## Figures and Tables

**Table 1:** Group characteristics.

Variable	Phaco-Canaloplasty *n* = 47	Phaco-iStent *n* = 45	MD/OR 95% CI	*p*
Sex (women), *n* (%)	27 (57.4)	35 (77.8)	2.59 (1.04, 6.44)	0.038
Age, M ± SD	65.11 ± 13.67	72.35 ± 8.00	7.24 (2.49, 11.99)	0.0032
Medications (baseline), *n* (%)				
0	1 (2.1)	2 (5.7)	-	0.406
1	11 (23.4)	13 (37.1)		
2	18 (38.3)	10 (28.6)		
≥3	17 (36.2)	10 (28.6)		
BCVA (baseline)	0.80 (0.50, 1.00)	0.60 (0.40, 1.00)	−0.20 (−0.20, 0.00)	0.456
IOP (baseline), *n* (%)				
≥18 and ≤21	13 (59.1)	9 (50.0)	1.44 (0.41, 5.07)	0.750
>21	9 (40.9)	9 (50.0)		
IOP (baseline)	17.30 (15.00, 19.85)	17.50 (15.10, 20.90)	0.20 (−1.60, 1.90)	0.876
ECD (baseline)	2309.50 (2032.00, 2533.00)	2160.00 (1958.50, 2372.50)	−149.50 (−296.00, 39.00)	0.116
Follow-up time (months), M ± SD	4.81 ± 3.16	5.28 ± 3.74	0.47 (−0.96, 1.90)	0.5162
LOCS III scale (NC 1/NC 2/NC 3)	14/28/5	13/27/5	-	0.899

MD/OR with 95% CI: median difference/odds ratio with 95% confidence interval. Dependencies between groups and qualitative variables were determined using the chi-squared test or Fisher’s exact test. Quantitative variables between groups were compared using Student’s *t*-test or Mann–Whitney U test.

**Table 2:** Comparison of BCVA according to type of surgery: baseline vs. further measurements.

Variable	Phaco-Canaloplasty *n* = 47	Phaco-iStent *n* = 45	MD/OR 95% CI	*p*
BCVA (baseline)	0.80 (0.50, 1.00)	0.60 (0.40, 1.00)	−0.20 (−0.20, 0.00)	0.456
BCVA (after 3 months)	1.00 (0.65, 1.00)	1.00 (0.90, 1.00)	0.00 (0.00, 0.10)	0.375
BCVA (after 6 months)	0.95 (0.60, 1.00)	1.00 (1.00, 1.00)	0.05 (0.00, 10.00)	0.037

MD/OR with 95% CI: median difference/odds ratio with 95% confidence interval. Dependencies between groups and qualitative variables were determined using the chi-squared test or Fisher’s exact test. Quantitative variables between groups were compared using Student’s *t*-test or Mann–Whitney U test.

**Table 3:** Comparison of ECD according to type of surgery: baseline vs. further measurements.

Variable	Phacocanaloplasty *n* = 47	Phaco-iStent *n* = 45	MD/OR 95% CI	*p*
ECD (baseline)	2309.50 (2032.00, 2533.00)	2160.00 (1958.50, 2372.50)	−149.50 (−296.00, 39.00)	0.116
ECD (after 3 months)	1878.38 ± 564.69	1440.66 ± 589.33	−437.72 (−733.49, 141.95)	0.0042
ECD (after 6 months)	1966.50 (1262.00, 2353.50)	1231.00 (1089.00, 2050.00)	−735.50 (−784.00, −27.00)	0.037

MD/OR with 95% CI: median difference/odds ratio with 95% confidence interval. Dependencies between groups and qualitative variables were determined using the chi-squared test or Fisher’s exact test. Quantitative variables between groups were compared using Student’s *t*-test or the Mann–Whitney U test.

**Table 4:** Comparison of IOP according to type of surgery: baseline vs. further measurements.

Variable	Phacocanaloplasty *n* = 47	Phaco-iStent *n* = 45	MD/OR 95% CI	*p*
IOP (baseline)	17.30 (15.00, 19.85)	17.50 (15.10, 20.90)	0.20 (−1.60, 1.90)	0.876
IOP (after 3 months)	14.84 ± 2.86	14.94 ± 2.72	0.10 (−1.32, 1.50)	0.8972
IOP (after 6 months)	15.00 (13.00, 16.00)	15.00 (14.00, 17.00)	0.00 (−1.00, 2.00)	0.438

MD/OR with 95% CI: median difference/odds ratio with 95% confidence interval. Dependencies between groups and qualitative variables were determined using the chi-squared test or Fisher’s exact test. Quantitative variables between groups were compared using Student’s *t*-test or Mann–Whitney U test.

**Table 5:** Comparison of antiglaucoma medications according to type of surgery–baseline vs. further measurements.

Variable	Phacocanaloplasty *n* = 47	Phaco-iStent *n* = 45	MD/OR 95% CI	*p*
Medications (baseline), *n* (%)				
0	1 (2.1)	2 (5.7)	-	0.406
1	11 (23.4)	13 (37.1)		
2	18 (38.3)	10 (28.6)		
≥	17 (36.2)	10 (28.6)		
Medications (after 3 months), *n* (%)				
0	27 (84.3)	28 (90.3)	-	0.7711
1	2 (6.3)	0 (0.0)		
2	2 (6.3)	2 (6.5)		
≥3	1 (3.1)	1 (3.2)		
Medications (after 6 months), *n* (%)				
0	22 (78.6)	29 (96.7)	-	0.0891
1	3 (10.7)	0 (0.0)		
2	2 (7.1)	1 (3.3)		
≥3	1 (3.6)	0 (0.0)		

MD/OR with 95% CI: median difference/odds ratio with 95% confidence interval. Dependencies between groups and qualitative variables were determined using the chi-squared test or Fisher’s exact test. Quantitative variables between groups were compared using Student’s *t*-test or Mann–Whitney U test.

**Table 6:** Comparison of selected characteristics: baseline vs. further measurements (PC group).

Variable (Baseline vs.)	Baseline	Further Measurement	MD (95% CI)	*p*
	Me (Q1, Q3)/M ± SD or *n* (%)			
ECD				
after 14 days, *n* = 33	2445.00 (2187.00, 2598.00	2251.00 (1842.00, 2358.00)	−106.00 (−374.00, −74.00)	<0.001
after 1 month, *n* = 31	2317.00 (2109.50, 2565.50)	2065.00 (1607.00, 2335.50)	−252.00 (−334.00, −149.50)	<0.001
after 3 months, *n* = 32	2254.72 ± 335.42	1878.38 ± 564.69	−376.34 (−538.36, −214.32)	<0.0011
after 6 months, *n* = 28	2309.50 (1975.00, 2499.00)	1966.50 (1262.00, 2353.50)	−313.00 (−550.00, −172.00)	<0.001
Visus				
after 14 days, *n* = 37	0.70 (0.50, 1.00)	0.80 (0.60, 1.00)	0.10 (0.00, 0.10)	0.278
after 1 month, *n* = 35	0.70 (0.45, 1.00)	0.90 (0.60, 1.00)	0.20 (0.00, 0.15)	0.039
after 3 months, *n* = 32	0.75 (0.45, 1.00)	1.00 (0.65, 1.00)	0.25 (0.00, 0.20)	0.037
after 6 months, *n* = 28	0.80 (0.55, 1.00)	0.95 (0.60, 1.00)	0.15 (0.00, 0.10)	0.088
IOP				
after 14 days, *n* = 37	18.00 (15.00, 20.10)	15.00 (12.00, 18.00)	−3.00 (−4.20, −1.30)	<0.001
after 1 month, *n* = 35	17.00 (15.00, 20.55)	14.00 (11.50, 16.50)	−3.00 (−5.20, −2.05)	<0.001
after 3 months, *n* = 32	17.80 (15.05, 21.20)	14.50 (13.00, 17.00)	−3.30 (−5.35, −2.25)	<0.001
after 6 months, *n* = 27	18.00 (15.35, 20.50)	15.00 (13.00, 16.00)	−3.00 (−5.35, −2.00)	<0.001
IOP ≤ 15 mmHg				
after 14 days, *n* = 37	10 (27.0)	20 (54.1)	-	0.031
after 1 month, *n* = 35	11 (31.4)	22 (62.9)	-	0.013
after 3 months, *n* = 32	8 (25.0)	20 (62.5)	-	0.004
after 6 months, *n* = 27	7 (25.9)	18 (66.7)	-	0.003
IOP ≤ 18 mmHg				
after 14 days, *n* = 37	19 (51.4)	29 (78.4)	-	0.013
after 1 month, *n* = 35	20 (57.1)	32 (91.4)	-	0.002
after 3 months, *n* = 32	18 (56.3)	29 (90.6)	-	0.007
after 6 months, *n* = 27	15 (55.6)	24 (88.9)	-	0.035
ACD				
after 14 days, *n* = 17	3.30 ± 0.56	4.14 ± 0.69	0.85 (0.37, 1.31)	0.0021
after 1 month, *n* = 16	3.33 ± 0.54	4.02 ± 0.64	0.69 (0.32, 1.05)	0.0011
after 3 months, *n* = 14	3.14 (3.01, 3.48)	4.52 (3.38, 5.10)	1.38 (0.96, 1.71)	0.030
after 6 months, *n* = 9	3.14 ± 0.31	4.28 ± 0.92	1.14 (0.41, 1.87)	0.0071
Medications				
after 14 days, *n* = 37				
0	1 (2.7)	37 (100.0)	-	<0.001
1	7 (18.9)	0 (0.0)		
2	16 (43.2)	0 (0.0)		
3+	13 (35.1)	0 (0.0)		
after 1 month, *n* = 35				
0	4 (11.4)	33 (94.3)	-	<0.001
1	8 (22.9)	0 (0.0)		
2	14 (40.0)	2 (5.7)		
3+	9 (25.7)	0 (0.0)		
after 3 months, *n* = 32				
0	4 (12.5)	27 (84.4)	-	<0.001
1	7 (21.9)	2 (6.3)		
2	11 (34.4)	2 (6.3)		
3+	10 (31.3)	1 (3.1)		
after 6 months, *n* = 28				
0	4 (14.3)	22 (78.6)	-	<0.001
1	4 (14.3)	3 (10.7)		
2	10 (35.7)	2 (7.1)		
3+	10 (35.7)	1 (3.6)		

Quantitative variables are presented as median with quartiles 1 and 3 or mean with standard deviation, and qualitative variables are presented as *n* (%). Comparisons of quantitative characteristics between measurements were performed using Wilcoxon’s test or Student’s *t*-test for dependent samples. Dependencies between measurements and qualitative variables with two categories or more than two categories were analysed using McNemar’s test and Wilcoxon’s test, respectively.

**Table 7:** Comparison of selected characteristics: baseline vs. further measurements (PiS group).

Variable (Baseline vs.)	Baseline	Further Measurement	MD (95% CI)	*p*
	Me (Q1; Q3)/M ± SD or *n* (%)			
ECD				
after 14 days, *n* = 21	2142.81 ± 423.32	1448.00 ± 716.52	−694.81 (−966.95, −422.67)	<0.001 ^1^
after 1 month, *n* = 19	2160.00 (2019.50, 2318.00)	1269.00 (895.00, 1806.50)	−891.00 (−1009.00, −512.00)	<0.001
after 3 months, *n* = 29	2178.79 ± 445.08	1440.66 ± 589.33	−738.14 (−912.11, −564.18)	<0.001 ^1^
after 6 months, *n* = 29	2296.00 (2063.00, 2420.00)	1231.00 (1089.00, 2050.00)	−1065.00 (−955.50, −528.00)	<0.001
BCVA				
after 14 days, *n* = 24	0.65 (0.35; 1.00)	0.90 (0.70, 1.00)	0.25 (0.00, 0.30)	0.031
after 1 month, *n* = 21	0.60 (0.30; 1.00)	1.00 (0.70, 1.00)	0.40 (0.05, 0.40)	0.006
after 3 months, *n* = 29	0.70 (0.50; 1.00)	1.00 (0.90, 1.00)	0.30 (0.05, 0.35)	0.002
after 6 months, *n* = 29	0.60 (0.50; 1.00)	1.00 (1.00, 1.00)	0.40 (0.10, 0.35)	0.001
IOP				
after 14 days, *n* = 24	16.90 (14.55; 19.85)	17.50 (15.00, 19.50)	0.60 (−2.05, 2.40)	0.820
after 1 month, *n* = 21	17.00 (15.00; 19.00)	15.00 (14.00, 16.00)	−2.00 (−3.95, 0.50)	0.130
after 3 months, *n* = 29	17.42 ± 3.77	14.79 ± 2.64)	−2.63 (−4.38, −0.88)	0.005 ^1^
after 6 months, *n* = 29	17.40 (15.10; 19.00)	15.00 (14.00, 16.00)	−2.40 (−3.30, −0.85)	0.002
IOP ≤ 15 mmHg				
after 14 days, *n* = 24	6 (25.0)	8 (33.3)	-	0.727
after 1 month, *n* = 21	6 (28.6)	11 (52.4)	-	0.180
after 3 months, *n* = 29	8 (27.6)	18 (62.1)	-	0.013
after 6 months, *n* = 29	7 (24.1)	18 (62.1)	-	0.003
IOP ≤ 18 mmHg				
after 14 days, *n* = 24	16 (66.7)	16 (66.7)	-	>0.999
after 1 month, *n* = 21	15 (71.4)	17 (81.0)	-	0.727
after 3 months, *n* = 29	19 (65.5)	25 (86.2)	-	0.109
after 6 months, *n* = 29	19 (65.5)	26 (89.7)	-	0.016
ACD				
after 14 days, *n* = 17	2.86 (2.68, 3.14)	4.08 (3.94, 4.68)	1.22 (1.10, 1.63)	<0.001
after 1 month, *n* = 14	2.98 (2.82, 3.23)	4.66 (4.08, 5.41)	1.76 (1.10, 1.96)	<0.001
after 3 months, *n* = 22	2.87 (2.68, 3.15)	4.88 (4.19, 5.20)	2.01 (1.42, 2.15)	<0.001
after 6 months, *n* = 23	2.86 (2.70, 3.15)	5.02 (4.17, 5.23)	2.16 (1.40, 2.21)	<0.001
Medications				
after 14 days, *n* = 20				
0	1 (5.0)	20 (100.0)	-	<0.001
1	6 (30.0)	0 (0.0)		
2	7 (35.0)	0 (0.0)		
3+	6 (30.0)	0 (0.0)		
after 1 month, *n* = 17				
0	2 (11.8)	16 (94.1)	-	<0.001
1	5 (29.4)	0 (0.0)		
2	5 (29.4)	1 (5.9)		
3+	5 (29.4)	0 (0.0)		
after 3 months, *n* = 23				
0	2 (8.7)	21 (91.3)	-	<0.001
1	8 (34.8)	0 (0.0)		
2	7 (30.4)	2 (8.7)		
3+	6 (26.1)	0 (0.0)		
after 6 months, *n* = 22				
0	2 (9.1)	21 (95.5)	-	<0.001
1	8 (36.4)	0 (0.0)		
2	6 (27.3)	1 (4.5)		
3+	6 (27.3)	0 (0.0)		

Quantitative variables are presented as median with quartiles 1 and 3 or mean with standard deviation, and qualitative variables are presented as *n* (%). Comparisons of quantitative characteristics between measurements were performed using Wilcoxon’s test or Student’s *t*-test ^1^ for dependent samples. Dependencies between measurements and qualitative variables with two categories or more than two categories were analysed using McNemar’s test and Wilcoxon’s test, respectively.

## Data Availability

All materials and information are available upon an e-mail request to the corresponding author.
